# Serious Games Are Not Serious Enough for Myoelectric Prosthetics

**DOI:** 10.2196/28079

**Published:** 2021-11-08

**Authors:** Christian Alexander Garske, Matthew Dyson, Sigrid Dupan, Graham Morgan, Kianoush Nazarpour

**Affiliations:** 1 Intelligent Sensing Laboratory School of Engineering Newcastle University Newcastle upon Tyne United Kingdom; 2 Edinburgh Neuroprosthetics Laboratory School of Informatics University of Edinburgh Edinburgh United Kingdom; 3 Networked and Ubiquitous Systems Engineering Group School of Computing Newcastle University Newcastle upon Tyne United Kingdom

**Keywords:** rehabilitation, serious games, engagement, transfer, upper limb, arm prosthesis, virtual training, virtual games

## Abstract

Serious games show a lot of potential for use in movement rehabilitation (eg, after a stroke, injury to the spinal cord, or limb loss). However, the nature of this research leads to diversity both in the background of the researchers and in the approaches of their investigation. Our close examination and categorization of virtual training software for upper limb prosthetic rehabilitation found that researchers typically followed one of two broad approaches: (1) focusing on the game design aspects to increase engagement and muscle training and (2) concentrating on an accurate representation of prosthetic training tasks, to induce task-specific skill transfer. Previous studies indicate muscle training alone does not lead to improved prosthetic control without a transfer-enabling task structure. However, the literature shows a recent surge in the number of game-based prosthetic training tools, which focus on engagement without heeding the importance of skill transfer. This influx appears to have been strongly influenced by the availability of both software and hardware, specifically the launch of a commercially available acquisition device and freely available high-profile game development engines. In this Viewpoint, we share our perspective on the current trends and progress of serious games for prosthetic training.

## Background

Adherence of patients to interventions (eg, home exercises) remains a key challenge in rehabilitation medicine [1]. Patients complain that exercises often feel tedious and tiring and that progress, if any, is slow and incremental [1]. Delivering virtual training in the form of games can help overcome issues related to nonadherence (or noncompliance) of patients to their exercise regimen [1]. The use of serious games has been recommended to motivate patients in performing their prescribed exercises consistently and completely [2-4].

The stroke rehabilitation literature includes a large number of publications that use serious games. Koutisiana et al [5] identified 96 publications between the years 1999 and 2019. The serious games used in stroke rehabilitation are showing significant benefits for the users, most notably an increased number of repetitions performed, which is a prime goal for this kind of rehabilitation [6]. Supported by this academic evidence, rehabilitation programs like Rehability (Imaginary srl), which has grown out of the Rehab@Home project [7], are being incorporated in clinical practice.

Although serious games have found their way into a multitude of areas of everyday life, industry, and research, including prosthetic training [8], academic results supporting the efficacy of serious games in myoelectric prosthetic training are scarce, if not nonexistent. Using games in virtual rehabilitation has been a part of research for 30 years [9], but they have only gained proper traction in the field in the last decade. This rise coincided with the commercialization of a range of game-related technologies (eg, motion tracking cameras and game controllers with inertial measurement sensors [6]). In this paper, we will offer our perspective on the efficacy of virtual training in general and serious games specifically for myoelectric prosthetics training.

Current research claims that the use of serious games in myoelectric prosthetics training has promise to improve training. Examples include faster learning [10], reduction of fatigue and irritation while training [11], and increased muscle control [12]. In addition, serious games can offer a faster route to myoelectric training after limb loss [11], as a game would likely not rely on socket fitting or full wound closure. Furthermore, it can make the training more enjoyable and engaging [11], as well as affordable and accessible for the home environment [10]. It also has the potential to assist the user with their body image [11], decrease phantom limb pain [11], and let the user feel more in charge of their own rehabilitation [8,10], while at the same time make it feel less like rehabilitation [8].

The prevailing view is that this combination of positive effects has the potential to significantly add to the existing prosthetic training and lead to a reduction in prosthesis abandonment, which has been linked to a lack of motivation and engagement [13] and poor training [10]. The performance of virtual prosthetic training at home can also offer benefits to the therapists. As a supplement to existing training regimes, it can offer an objective measure of how diligently the patient is doing their exercises at home and of their improvements [10]. It also has the potential to decrease rehabilitation times and the time necessary for each patient, thereby reducing the workload for therapists [8].

We investigated papers that included any virtual training or assessment for upper limb prosthesis control using myoelectric signals as input. The included papers were identified during investigation of the literature and has been augmented with systematic searches in multiple databases, including PubMed, Web of Science, and Google Scholar. This led to the inclusion of 55 journal articles and conference papers, with a total of 59 different virtual training programs. CAG classified these programs into two categories, namely serious games and simulators (Table 1), according to Narayanasamy et al [14]. Both training simulators and serious games are interactive simulations in a virtual environment with the purpose of skill development. Simulators often duplicate real-world scenarios, require standard operational procedures, are not designed for entertainment, have no secondary purpose, and usually do not have an obvious final state. Conversely, a serious game is set in a fictitious scenario, provides various challenges, allows for entertainment, and allows the user to develop gameplay patterns while trying to achieve game-specific goals. This can include an end state. Therefore, some of the programs are classified as “simulators,” even when the authors identified them as “games.” The programs were further classified by the type of task the user was given, the type of control scheme the program used, and the input and output devices that were used. This more detailed table can be found in Multimedia Appendix 1.

**Table 1:** Categorization of the virtual training programs.

Names			Publications
**Serious games**			
	Air-Guitar Hero (rhythm game)	[15]	
	WiiEMG (sports game)	[16]	
	Sonic Racing (racing game)	[17]	
	MyoBox (dexterity game)	[18,19]	
	Flappy Bird (sidescroller)^a^	[20]	
	Space Invaders (fixed shooter)^a^	[20]	
	MyoBeatz (rhythm game)^b^	[21]	
	Falling of Momo (vertical scroller)^b^	[22-25]	
	Volcanic Crush (reaction game)^a,b^	[10]	
	Dino Sprint (endless runner)^a,b^	[10]	
	Dino Feast (dexterity game)^a,b^	[10]	
	Space ARMada (fixed shooter)	[11]	
	SuperTuxKart (racing game)	[2,12,26,27]	
	Step Mania 5 (rhythm game)	[2,12,26,27]	
	Pospos (dexterity game)	[2,12,26,27]	
	Who nose?/Nose Picker (simple game)^a^	[28,29]	
	Smash Bro/Bash and Debris (sidescroller)^a^	[28,29]	
	Sushi Slap (action game)^a^	[28,29]	
	Crazy Meteor (multidirectional shooter)^a^	[28,29]	
	Dog Jump/Beeline Border Collie (sidescroller)	[28,29]	
	Breakout-EMG (arcade game)	[30]	
	Training Game Prototype^a^	[31]	
	Dino Claw (dexterity game)^a,b^	[10]	
	Training, TAC^c^ test, and Crossbow Game^a^	[32]	
	UpBeat (rhythm game)^a,b^	[4]	
	Rhythm Game^a,b^	[13]	
	Crate Whacker (tech demo)^b^	[33]	
	Race the Sun (endless runner)^b^	[33]	
	Fruit Ninja (dexterity game)^b^	[33]	
	Kaiju Carnage (action game)^b^	[33]	
**Simulators**			
	UVa Neuromuscular Training System	[34,35]	
	Commercial software PAULA	[36]	
	Virtual training	[36]	
	Virtual training environment	[37]	
	Mixed reality training^b^	[38]	
	Virtual box and beans test^b^	[39]	
	Virtual box and blocks test^a,b^	[40]	
	Virtual rehabilitation training tool^a,b^	[41]	
	VITA: Virtual Therapy Arm^a,b^	[42]	
	Exploration^a,b^	[43]	
	AR prosthesis simulator	[44]	
	Virtual training system	[45-47]	
	Training system	[48]	
	Catching simulator	[49]	
	Performance assessment	[50]	
	Catching simulator Prosthesis Gripper	[19]	
	MSMS (Musculoskeletal Modelling Software)	[51,52]	
	Prosthesis simulator	[53]	
	VR^d^ testing environment	[54]	
	Virtual simulation	[55]	
	VR evaluation environment	[56]	
	Virtual reality environment System	[57]	
	AR^e^ training system^a^	[58,59]	
	Myoelectric training tool	[60]	
	Training environment	[61]	
	Virtual prosthesis	[62]	
	Virtual model	[63]	
	Training platform	[64]	
	Manus VR Training Platform	[65]	
	Dual-arm EMG^f^ signal control training system	[66]	
	Myoelectric Control Evaluation and Trainer System	[67]	

^a^Developed using the Unity engine.

^b^Uses the Myo Gesture Control Armband.

^c^TAC: Target Achievement Control.

^d^VR: Virtual Reality.

^e^AR: Augmented Reality.

^f^EMG: electromyography.

## Different Approaches

**Figure 1:**
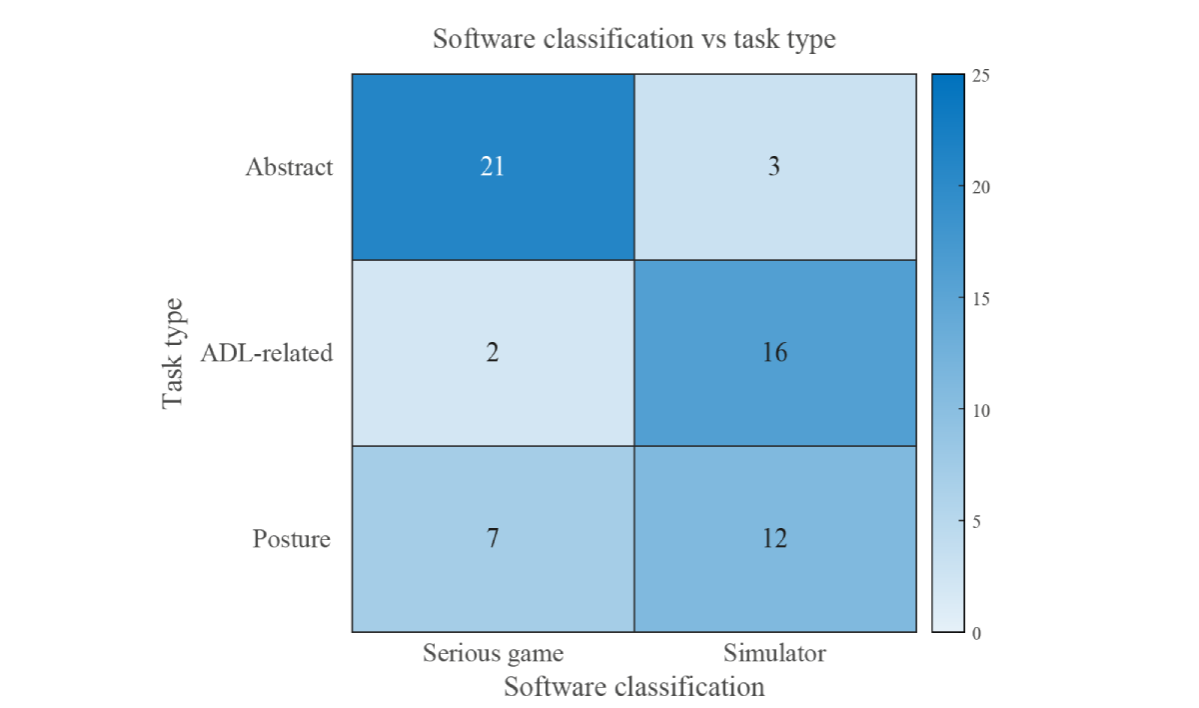
Heatmap of the authors’ software classification against the performed tasks: serious games using abstract tasks [2,10-12,15-30], tasks related to ADL [10,31], and posture reproduction tasks [4,13,32,33]; as well as simulators using abstract tasks [34-36], ADL-related tasks [19,37-54], and posture reproduction tasks [56-67]. ADL: activities of daily living.

The categorization of the publications in this field and the software presented therein has shown a significant split of the approaches of researchers into roughly two groups, as can be seen in Figure 1. This divide is most noticeable with regard to whether the software is classified as a game or a simulator and which type of tasks are implemented. The first approach focuses on the engagement and motivation of the user and seems to have grown in popularity in recent years. Researchers develop serious games that often have an explicit or implicit focus on game design elements to keep the user engaged in the game and therefore in the rehabilitation or training. The majority of these myogames (21/30) incorporate abstract tasks not resembling a real-life scenario. These games attempt to train the user in the use of a myoelectric prosthesis by focusing on different aspects of muscle control, including proportional control, independent control, and others. Only two games feature a task that is somewhat activities of daily living–related, both consisting of variants of a pick-and-place task, one stationary [10] and one moving in a 3D environment [31]. In a further seven games, the user is tasked with reproducing specific postures in two rhythm games [4,13], a virtual reality crossbow game [32], and four open-source games [33].

Publications introducing and assessing these games show that they are engaging and enjoyable to the participants. Some studies involving people with limb difference showed their willingness to use them in a home environment [8,21,27]. With regard to skill acquisition, a general increase in in-game performance is shown for a number of these games [12,25,26]. However, it was rarely tested whether these myogames increase prosthetic ability or speed up the learning process of acquiring that skill. The one research group that tested for an increase in prosthetic ability did not find evidence of a significant increase following the playing of a myogame for different control schemes [19,30].

The second approach focuses on skill transfer and therefore involves the development and investigation of simulators that mostly show the user a representation of the real world and require the performance of activities of daily living–related or posture reproduction tasks. Only two training programs that were classified as simulators used abstract tasks; these tasks were embedded in a sterile software environment and lacked distinctive game traits. In this type of research, the focus is on the effectiveness of the skill transfer from the virtual training to actual prosthetic ability. The prescribed tasks can involve recreations or tasks inspired by tests used in the assessment of prosthetic ability, like the Southampton Hand Assessment Procedure (SHAP) test [68], the Target Achievement Control (TAC) test [69], and others. The focus on task specificity for learning prosthetic skills seems like a promising approach as the results of one study indicated that skill transfer occurred. Performing a virtual task resembling the control of a prosthetic hand led to an increase in prosthetic ability [49] as opposed to when the task was to play a classic arcade game [30]. The task specificity therefore seems to have an influence on the effectiveness of virtual training; however, further research must be done to substantiate this.

The effectiveness of virtual training in increasing prosthetic ability is without doubt one of the necessary requirements for any adoption into clinical rehabilitation; however, consensus on a universal measure of effectiveness is not available. A criticism of the myogames in the game-focused group is that they work on the implied assumption that an improvement in skill performing any myoelectric task will lead to an improvement in prosthetic skill [49]. Although it has been shown that the user increases their skill in different aspects on the muscular level [10,25], it is not clear whether that influences the way or speed at which a person might acquire prosthetic skill.

## Other Influences

**Figure 2:**
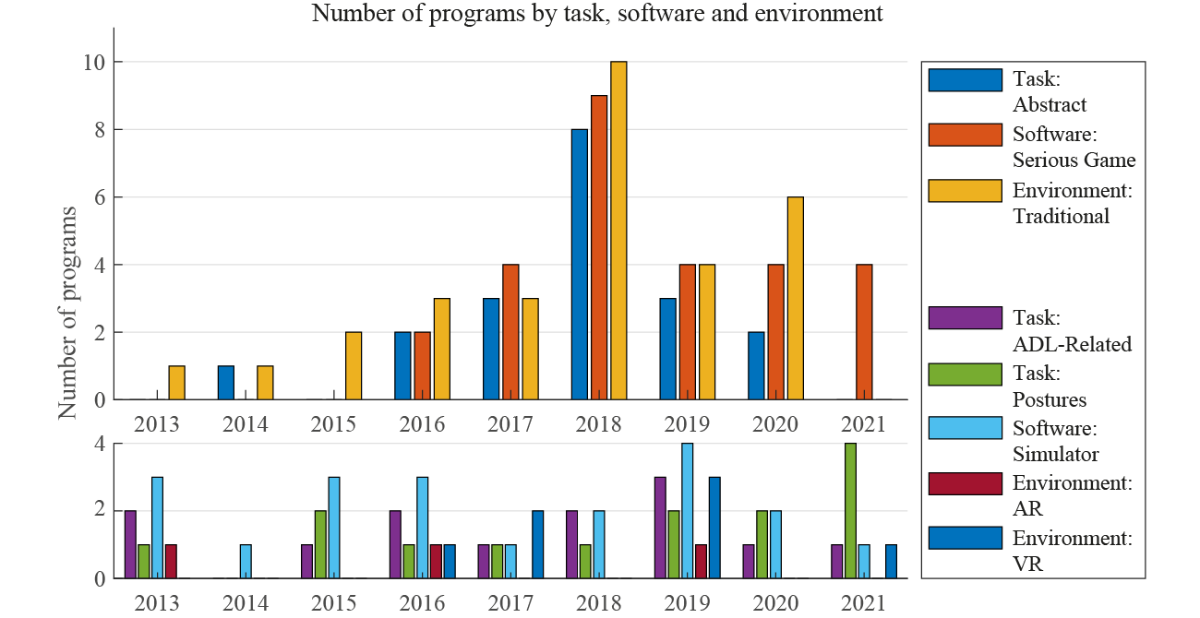
Number of training software introduced by task, software, and environment type. ADL: activities of daily living; AR: augmented reality; VR: virtual reality.

Figure 2 shows another interesting development regarding the first research approach. It clearly features a joint spike in more recent years in the development of serious games incorporating an abstract task and presented in a nonimmersive environment using traditional media. The development of simulators and software using other task types and environment experiences has remained comparatively steady over the same time frame. The start of this spike in publications coincides with the release of the Myo Gesture Control Armband (Thalmic Labs), a dry surface electrode armband, on the commercial market in the year 2015 [70]. The spike in publications started to decrease when the company stopped selling this product in 2018. However, even though it is no longer sold, the Myo armband is still in widespread use in research as there is currently no commercial alternative available. In total, 30% of the programs analyzed use the Myo armband and the most recent work using it was published in 2021 [61]. The uptake in development has likely been further boosted due to a few professional commercial game development engines being free for personal and low-profit use alongside the provision of an application programming interface (API) for the Myo Armband; there are numerous open-source examples of the use of this API in these engines. The main example of one of these engines is the Unity game engine (Unity Technologies) made freely available in the year 2009 [71]; this game engine has been used in at least one-third of the published programs.

These two factors indicate that the development of serious games intended for prosthetic training was strongly influenced by the emergence of readily available software and hardware technology. However, the enthusiastic embrace of the newly available technology tended toward research exploring the engagement aspects of game design. This is likely due to the low barrier to entry of this approach as there are a multitude of resources for game development available and the study of engagement does not require the involvement of people with limb difference. Investigation of these serious games confirmed that people are more willing to engage in learning a task if it is an intrinsically enjoyable and motivating experience [2]. Such research has also shown that with these games, participants are able to quickly master fine control of their muscles [10,25]. However, this research often tacitly assumed an efficacy in skill transfer by this virtual muscle training, which has yet to be substantiated. As such, it is not clear whether this increase in motor control would lead to enhanced prosthesis control and which types of games might be more conducive to learning how to use a prosthetic device. Therefore, at this point in time, serious games are not serious enough to train upper limb prosthesis use effectively.

## Where Do We Go From Here?

In the research targeting other conditions, such as stroke rehabilitation, the main target is to get the user to move their respective body part more to regain a substantial degree of control over it. The reason for the strong focus on the engagement and motivation of users to increase repetitions of a movement is therefore clear. However, using a myoelectric upper limb prosthesis requires the user to acquire a completely new set of skills. This can mean to either retrain or newly train muscles and their associated uses, depending on whether the limb difference is acquired or congenital. Therefore, a necessary requirement for a serious game in this field to be considered for clinical adoption would be evidence of a benefit to prosthetic ability (ie, evidence that the skill learned in the game transfers to the use of an actual myoelectric prosthesis). So far this kind of skill transfer has only been shown for software that we classified as simulators. It is hypothesized that the task specificity of the actions performed virtually allows the transfer to the real world to occur [49].

**Figure 3:**
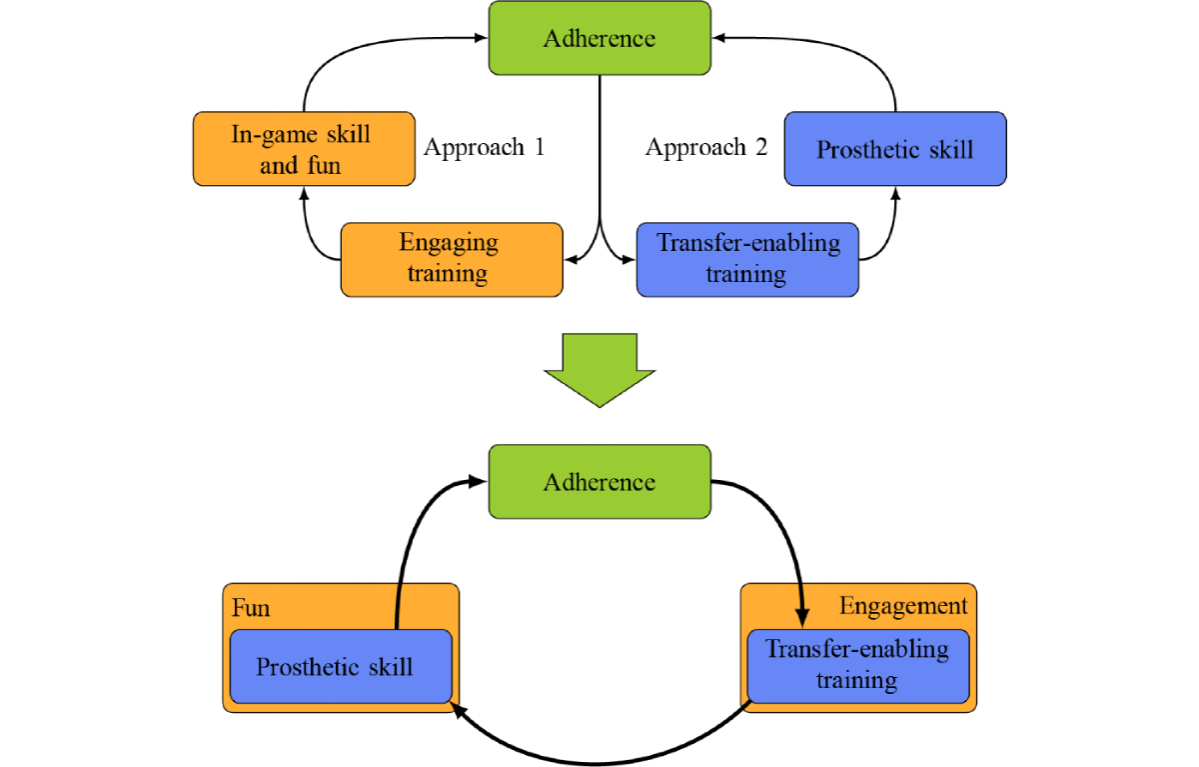
Diagram of the existent and recommended habit loops.

Research in this field needs to establish viable paths for transfer to occur before focusing on the topic of engaging and motivating the target user group. Serious games intended for prosthetic training need to show their benefit for prosthetic ability, be it direct or indirect. Hence, a sensible approach for the development of such a serious game could be to first demonstrate which types of tasks allow transfer at all and then to develop the engaging and motivating game structure around it. As with other conditions, researchers employ the attractiveness of games to actively engage users; however, the clinical benefit cannot be neglected or compromised. The two different approaches in this field encourage separate habit loops when they should merge and form a single loop more beneficial for the user, as shown in Figure 3. Engagement should not be viewed separately but in conjunction with transfer-enabling training to enhance the habit formation of the user to adhere to the training.

Therefore, the research should establish one or multiple tasks that either directly enable skill transfer to prosthetic use or show evidence of supporting the acquisition of prosthetic skill. Built on these tasks, an engaging and motivating platform should be implemented, which should enable users to increase their prosthetic skill while having fun. The positive reinforcement of the skill increase combined with the fun experienced while training should have a positive effect on adherence to the training and therefore on the long-term success of the intervention.

Furthermore, the effect of the introduction of myogames on therapists’ workloads should be determined; this is highly dependent on the nature of the training and whether there is a need for the direct involvement of the therapist, which could potentially result in a similar or even larger workload [72]. As this factor depends strongly on the design of the program, it emphasizes the importance of smart development including the input and feedback of all parties involved, including clinicians and therapists, to lead to a product that benefits everyone.

In conclusion, research on prosthetic training has confirmed that myoelectric skills can be acquired with serious games. However, for the development of a viable serious game intended for prosthetic training, it is necessary to validate the “serious” part of the game, namely the tasks that would allow for skill transfer. Serious games for prosthetic training can only be expected to yield fruitful results beyond engagement when they incorporate tasks that are found to facilitate prosthetic skill. We recommend that the research community investigates which types of myogame tasks might facilitate transfer, as the only existing results at the time of writing this paper indicate a lack of effectiveness [19,30,73]. This lack does not necessarily hold true for all tasks that are not related to activities of daily living, however, and ignoring abstract tasks entirely would exclude a wide range of possible avenues for prosthetic game development.

It would be beneficial to be more accurate regarding the terminology used in the field and, if the term “game” is used, to specify the incorporated game design elements explicitly. More long-term and ideally home-based experiments are needed to conclusively test for any prosthetic skill transfer that might occur with the consistent use of prosthetic gaming devices. Even though previous studies indicate that no change in prosthetic ability occurs after training with a myogame [19,30,73], these only tested the effect of comparatively short training sessions with able-bodied people or very small groups of prosthesis users. It should also be tested whether prosthetic gaming has the potential to support traditional prosthetic training by allowing for supplementary practice sessions between visits to the prosthetist.

## Appendix

Multimedia Appendix 1 Detailed categorization of the virtual training programs.

## Abbreviations

EMG: electromyography
SHAP: Southampton Hand Assessment Procedure
TAC: Target Achievement Control
