# Supplementary material for: Serious Games Are Not Serious Enough for Myoelectric Prosthetics
Source: JMIR Serious Games. 2021 Nov 8;9(4):e28079. doi: 10.2196/28079 (PMC8663510; doi:10.2196/28079)
Supplement: Multimedia Appendix 1 [file games_v9i4e28079_app1.pdf]

Table 1: Detailed categorization of the virtual training programs.

Program (Genre)	Type	Task	Input Device	Output Device	Control Strategy	Source/ Development	Publications
"Air-Guitar Hero" (Rhythm Game)	SG[a]	A[c]	C[f]	T[i]	PR[l]	Commercial	[15]
"WiiEMG" (Sports Game)	SG	A	C	T	PR	Commercial	[16]
"Sonic Racing" (Racing Game)	SG	A	C	T	D[m]	Commercial	[17]
"MyoBox" (Dexterity Game)	SG	A	C	T	PR	U	[18, 19]
"Flappy Bird" (Sidescroller)	SG	A	C	T	PR	Unity Engine	[20]
"Space Invaders" (Fixed Shooter)	SG	A	C	T	PR	Unity Engine	[20]
"MyoBeatz" (Rhythm Game)	SG	A	MB[g]	T	D	U	[21]
"Falling of Momo" (Vertical Scroller)	SG	A	MB	T	D	Processing	[22-25]
"Volcanic Crush" (Reaction Game)	SG	A	MB	T	D	Unity Engine	[10]
"Dino Sprint" (Endless Runner)	SG	A	MB	T	D	Unity Engine	[10]
"Dino Feast" (Dexterity Game)	SG	A	MB	T	D	Unity Engine	[10]
"Space ARMada" (Fixed Shooter)	SG	A	ME[h]	AR[j]	D	U	[11]
"SuperTuxKart" (Racing Game)	SG	A	ME	T	D	U	[2,12,26,27]
"Step Mania 5" (Rhythm Game)	SG	A	ME	T	D	U	[2,12,26,27]
"Pospos" (Dexterity Game)	SG	A	ME	T	D	U	[2,12,26,27]
"Who nose?"/ "Nose Picker" (Simple Game)	SG	A	ME	T	D	Unity Engine	[28,29]
"Smash Bro"/ "Bash and Debris" (Sidescroller)	SG	A	ME	T	D	Unity Engine	[28,29]
"Sushi Slap" (Action Game)	SG	A	ME	T	D	Unity Engine	[28,29]
"Crazy Meteor" (Multidirectional Shooter)	SG	A	ME	T	D	Unity Engine	[28,29]
"Dog Jump"/ "Beeline Border Collie" (Sidescroller)	SG	A	ME	T	D	Unity Engine	[28,29]

“Breakout-EMG” (Arcade Game)	SG	A	ME	T	D	U	[30]
Training Game Prototype	SG	ADL[d]	C	T	D	Unity Engine	[31]
“Dino Claw” (Dexterity Game)	SG	ADL	MB	T	D	Unity Engine	[10]
Training, TAC test, and Crossbow Game	SG	P[e]	C	VR[k]	PR	Unity Engine	[32]
“UpBeat” (Rhythm Game)	SG	P	MB	AR	PR	Unity Engine	[4]
Rhythm Game	SG	P	MB	T	PR	Unity Engine	[13]
“Crate Whacker” (Tech Demo)	SG	P	MB	T	PR	Commercial	[33]
“Race the Sun” (Endless Runner)	SG	P	MB	T	PR	Commercial	[33]
“Fruit Ninja” (Dexterity Game)	SG	P	MB	T	PR	Commercial	[33]
“Kaiju Carnage” (Action Game)	SG	P	MB	T	PR	Commercial	[33]
“UVa Neuromuscular Training System”	Sim[b]	A	ME	T	D	U	[34, 35]
Commercial Software “PAULA”	Sim	A	ME	T	D	Commercial	[36]
Virtual Training	Sim	A	ME	T	D	U	[36]
Virtual Training Environment	Sim	ADL	C	VR	PR	U	[37]
Mixed Reality Training	Sim	ADL	MB	AR	PR	U	[38]
Virtual Box and Beans Test	Sim	ADL	MB	T	PR	U	[39]
Virtual Box and Blocks Test	Sim	ADL	MB	VR	D	Unity	[40]
Virtual Rehabilitation Training Tool	Sim	ADL	MB	VR	PR	Unity Engine	[41]
“VITA: Virtual Therapy Arm”	Sim	ADL	MB	VR	PR	Unity Engine	[42]
Exploration	Sim	ADL	MB	VR	PR	Unity Engine	[43]
AR Prosthesis Simulator	Sim	ADL	ME	AR	PR	VRML[o] AR-ToolKit	[44]
Virtual Training System	Sim	ADL	ME	T	D	Virtools	[45-47]
Training System	Sim	ADL	ME	T	D	U	[48]
Catching Simulator	Sim	ADL	ME	T	D	U	[49]
Performance Assessment	Sim	ADL	ME	T	PR	Matlab	[50]

Catching Simulator “Prosthesis Gripper”	Sim	ADL	ME	T	PR	U	[19]
MSMS (“Musculoskeletal Modelling Software”)	Sim	ADL	ME	VR	D	Java	[51, 52]
Prosthesis Simulator	Sim	ADL	ME	VR	D	Gamestudio	[53]
VR Testing Environment	Sim	ADL	ME	VR	PR	Gamestudio	[54]
Virtual Simulation	Sim	N	ME	T	PR	VMRL, Java	[55]
VR Evaluation Environment	Sim	P	C	VR	PR	U	[56]
Virtual Reality Environment System	Sim	P	C	T	D	U	[57]
AR Training System	Sim	P	ME	AR	PR	Unity Engine	[58, 59]
Myoelectric Training Tool	Sim	P	ME	T	PR	Java	[60]
Training Environment	Sim	P	ME	T	PR	VRML	[61]
Virtual Prosthesis	Sim	P	ME	T	PR	OpenGL	[62]
Virtual Model	Sim	P	ME	T	PR	Matlab	[63]
Training Platform	Sim	P	ME	T	PR	Open Dynamics Engine, MSMS	[64]
“Manus VR Training Platform”	Sim	P	ME	T	PR	U	[65]
Dual-arm EMG signal control training system	Sim	P	ME	T	PR	U	[66]
“Myoelectric Control Evaluation and Trainer System”	Sim	P	ME	T	U[n]	Microsoft C5.1	[67]

Type: [a]Serious Game, [b]Simulator
Task: [c]Abstact, [d]Activities of Daily Living, [e]Posture reproduction
Input: [f]Custom Solution, [g]Thalmic Myo Gesture Control Armband, [h]Medical Electrodes
Output:[i]Traditional Media, [j]Augmented Reality, [k]Virtual Reality
Control: [l]Pattern Recognition, [m]Direct Control
[n]Unclear/Unspecified, [o]Virtual Reality Modeling Language
